# Endothelial Progenitor Cell-Based *in vitro* Pre-Endothelialization of Human Cell-Derived Biomimetic Regenerative Matrices for Next-Generation Transcatheter Heart Valves Applications

**DOI:** 10.3389/fbioe.2022.867877

**Published:** 2022-03-31

**Authors:** Sarah E. Motta, Polina Zaytseva, Emanuela S. Fioretta, Valentina Lintas, Christian Breymann, Simon P. Hoerstrup, Maximilian Y. Emmert

**Affiliations:** ^1^ Institute for Regenerative Medicine (IREM), University of Zurich, Zurich, Switzerland; ^2^ Wyss Translational Center Zurich, University and ETH Zurich, Zurich, Switzerland; ^3^ Department of Obstetrics and Gynaecology, University Hospital Zurich, Obstetric Research, Feto- Maternal Haematology Research Group, Zurich, Switzerland; ^4^ Department of Cardiovascular Surgery, Charité Universitätsmedizin Berlin, Berlin, Germany; ^5^ Department of Cardiothoracic and Vascular Surgery, German Heart Center Berlin, Berlin, Germany

**Keywords:** human cell-derived tissue-engineered matrices, endothelial colony forming cell, anti-coagulation, hemocomaptibility, scratch assay, HPL, transcatheter tissue-engineered valve, endothelial progenitor cell

## Abstract

Hemocompatibility of cardiovascular implants represents a major clinical challenge and, to date, optimal antithrombotic properties are lacking. Next-generation tissue-engineered heart valves (TEHVs) made from human-cell-derived tissue-engineered extracellular matrices (hTEMs) demonstrated their recellularization capacity *in vivo* and may represent promising candidates to avoid antithrombotic therapy. To further enhance their hemocompatibility, we tested hTEMs pre-endothelialization potential using human-blood-derived endothelial-colony-forming cells (ECFCs) and umbilical vein cells (control), cultured under static and dynamic orbital conditions, with either FBS or hPL. ECFCs performance was assessed *via* scratch assay, thereby recapitulating the surface damages occurring in transcatheter valves during crimping procedures. Our study demonstrated: feasibility to form a confluent and functional endothelium on hTEMs with expression of endothelium-specific markers; ECFCs migration and confluency restoration after crimping tests; hPL-induced formation of neo-microvessel-like structures; feasibility to pre-endothelialize hTEMs-based TEHVs and ECFCs retention on their surface after crimping. Our findings may stimulate new avenues towards next-generation pre-endothelialized implants with enhanced hemocompatibility, being beneficial for selected high-risk patients.

## Introduction

Thromboembolic events associated with currently available cardiovascular implants still remain a major problem in daily clinical routine. Thus, long-term anticoagulation or anti-platelet therapy is often required, which however permanently increases the risk for bleeding and decreases the overall patient’s quality of life. Therefore, a good hemocompatibility profile represents a key requirement in the development of novel cardiovascular implants (van Oeveren, 2013; Carnicelli et al., 2016).

Next-generation tissue-engineered (TE) replacements with regenerative capacities (Dahl et al., 2011; Weber et al., 2013; Driessen-Mol et al., 2014; Syedain et al., 2014; Syedain et al., 2015; Reimer et al., 2017; Syedain et al., 2017; Emmert et al., 2018; Lintas et al., 2018; Motta et al., 2018; Biermann et al., 2019; Boethig et al., 2019; Kirkton et al., 2019; Motta et al., 2019; Gerdisch et al., 2020; Fioretta et al., 2021; Syedain et al., 2021) have demonstrated their strong potential in numerous preclinical studies and first clinical pilot trials (Fioretta et al., 2021), and may therefore represent an ideal candidate to overcome the limitations of current prostheses. As one promising TE approach, we have recently introduced a biomimetic acellular tissue-engineered matrix (TEM), that is, manufactured from a polymer composite and an *in vitro* grown extracellular matrix (ECM), which can be engineered from different (human) cell sources (Emmert et al., 2018; Fioretta et al., 2021).

Importantly, prior to implantation, the TEM is decellularized leaving a cell-free construct which is thereby conceptually applicable to every patient (Dijkman et al., 2012). Preclinical evaluations of such decellularized TEMs in the context of heart valves and blood vessels have demonstrated their strong remodeling, and recellularization capacity, including continuous endothelialization over time (Weber et al., 2013; Driessen-Mol et al., 2014; Emmert et al., 2018; Lintas et al., 2018; Motta et al., 2018; Motta et al., 2019; Motta et al., 2020; Fioretta et al., 2021). As part of the remodeling process, the blood coagulation cascade is initiated immediately upon implantation of the TEM (Lynn et al., 2004; van Loon et al., 2013), where host cells are able to rapidly infiltrate to finally allow for the gradual formation of a functional endothelium, thereby ensuring a sufficient and safe performance of the TEM. However, the exact mechanisms of the endothelialization process (e.g., blood-borne and/or migration from the adjacent tissue) in such TEMs remain to be elucidated, especially as their endothelialization capacity may also be influenced by the patient’s individual regenerative potential. This aspect may be particularly relevant when using TEMs in the high-pressure circulation of the left heart (e.g., transcatheter aortic valve implantation, TAVI) which is known to carry a high risk for thromboembolic events.

Hence, from a translational perspective, the concept of TEM *in vitro* pre-endothelialization prior to implantation may be warranted for certain indications or specific patient populations with a high risk for thromboembolic events and may represent a good strategy to further enhance their overall hemocompatibility. Therefore, we here tested the hypothesis whether *in vitro* pre-endothelialization of human cell-based TEMs (hTEMs) using an endothelial progenitor cell (EPC)-based approach is feasible. We particularly focused on hTEM *in vitro* pre-endothelialization in the setting of transcatheter heart valves as these implants are prone to microstructural damage due to the required crimping maneuvers during delivery (Kiefer et al., 2011; Amahzoune et al., 2013; Alavi et al., 2014). Our group has previously developed several (h)TEM-based transcatheter valves (Weber et al., 2011; Weber et al., 2013; Driessen-Mol et al., 2014; Emmert et al., 2018; Lintas et al., 2018; Motta et al., 2018; Motta et al., 2019; Fioretta et al., 2021), and therefore we investigated if the integrity of an *in vitro* pre-endothelialized hTEM can also be preserved after such crimping procedures.

To test these hypotheses, we first evaluated the general *in vitro* pre-endothelialization capacity of EPC-seeded hTEMs in a high-throughput patch culture experiment. In a next step, we assessed the regenerative capacity of human blood-derived EPCs or human-derived umbilical vein endothelial cells (HUVECs) seeded hTEMs in a scratch assay experiment (Liang et al., 2007) under static and dynamic orbital conditions, which allowed us to mimic potential endothelial damages that may occur due to crimping of transcatheter valves. Thereafter, we generated hTEM-based transcatheter tissue engineered sinus valves (TESVs) which were then *in vitro* pre-endothelialized before they underwent crimping procedures to assess whether the endothelium remains stable. Finally, to enhance their translational relevance, the hTEM pre-endothelialization capacity was also assessed under xenogeneic-free culture conditions (i.e., supplemented with human platelet lysate (hPL)).

## Results

### Macroscopic Evaluation of Human Cell-Derived Tissue-Engineered Extracellular Matrices

**FIGURE 1:**
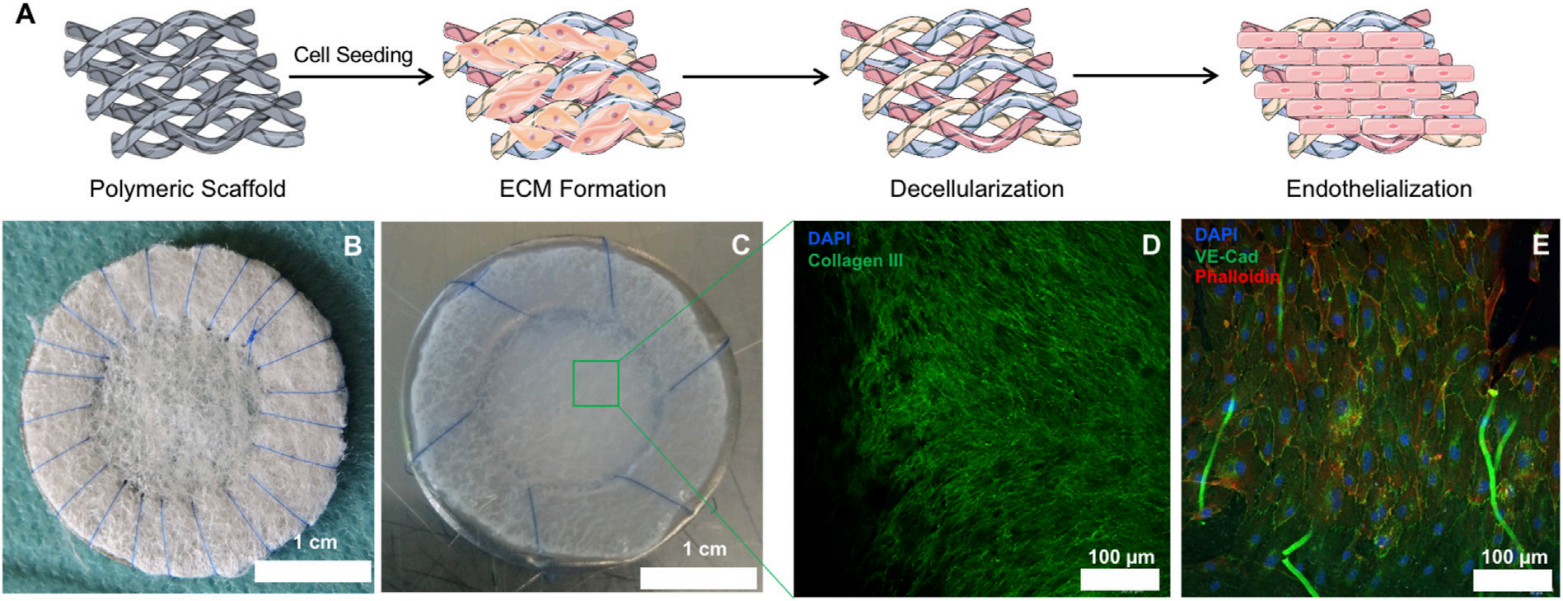
Macroscopic and microscopic appearance of hTEMs before and after *in vitro* culture. **(A)**: Schematic representation of the hTEM production procedure (Images adapted from Servier Medical Art under a creative common attribution 3.0 unported license). Briefly, PGA/P4HB scaffolds were sutured on stainless-steel metal ring and seeded with hDFs. After 4 weeks culture, patches were decellularized and finally endothelialized *in vitro*. **(B)**: Macroscopic image of the PGA/P4HB scaffold sutured onto a stainless-steel metal ring before cell seeding. **(C)**: Macroscopic appearance of the hTEM resulting from 4 weeks of tissue culture and subsequent decellularization. The hTEM is characterized by a shiny and smooth surface, indicating the presence of ECM. **(D)**: Confocal microscope image of the hTEM stained for collagen III (green) and DAPI (blue) reveals the presence of a dense and homogeneous collagenous matrix with complete absence of cell nuclei. **(E)**: Confocal microscope image showing homogeneous endothelialization on the hTEM surface.

Upon *in vitro* culture and decellularization of PGA/P4HB patches (Figures 1A,B), the resulting hTEMs displayed shiny and homogeneous ECM formation (Figure 1C). Newly formed tissue was evident in all hTEMs and confirmed by the expression of abundant Collagen III (Figure 1D). Successful decellularization procedure was confirmed by the absence of cell nuclei indicating a cell-free matrix (Figure 1D).

### Human Cell-Derived Tissue-Engineered Extracellular Matrix Pre-endothelialization Potential

**FIGURE 2:**
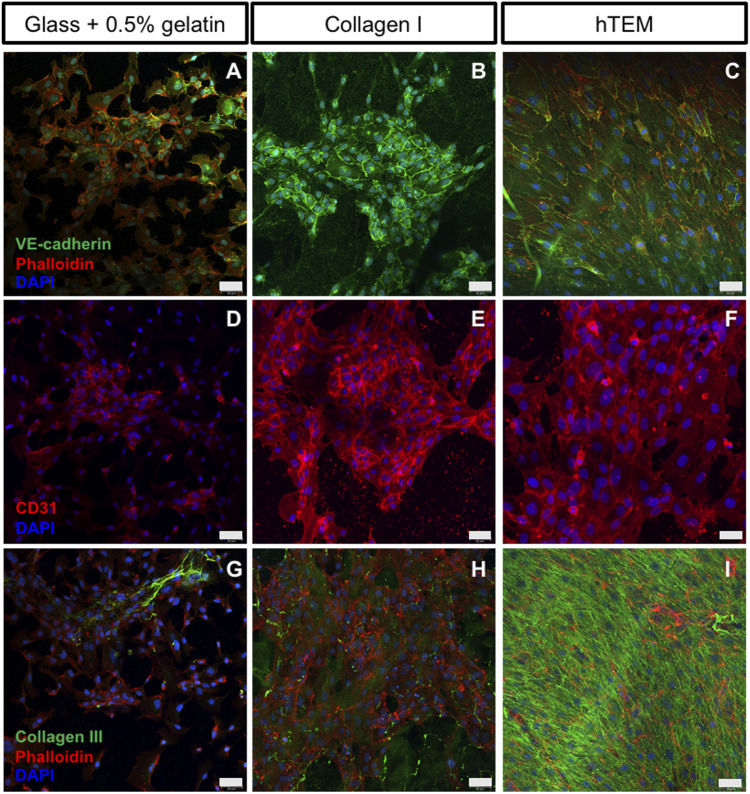
Confocal images of ECFCs cultured on gelatin-coated glass, collagen I gels, and hTEMs at 5 days. **(A–C)**: ECFCs stained for VE-cad (green), phalloidin (red), and DAPI (blue). **(D–F)**: ECFCs stained for CD31 (red) and DAPI (blue). **(G–I)**: Staining for Collagen III (green), phalloidin (red), and DAPI (blue). **(A,D,G)**: glass covered with 0.5% gelatin show limited pre-endothelialization potential with poor ECFCs confluency after 5 days of culture, limited cell-cell contact, and low expression of CD31 and VE-cad. **(B,E,H)**: Collagen I gel show spot-dependent pre-endothelialization. ECFCs are organized in colonies with presence of cell-cell contacts and expression of CD31 and VE-cad. **(C,F,I)**: ECFCs seeded onto hTEM substrates demonstrate good pre-endothelialization potential, by achieving complete confluency after 5 days of culture (50 µm scale bars).

hTEM pre-endothelialization potential of ECFCs and HUVECs (Figure 1E) was compared to two control substrates (0.5% gelatin-coated glass, and Collagen I gel) and assessed by immunofluorescence (Figure 2). ECFCs were characterized by flow cytometry (Supplementary Figure S1). After 5 days of ECFC culture, gelatin-coated glass (Figures 2A,D,G) and Collagen I substrates (Figures 2B,E,H) showed incomplete and inhomogeneous (spot-dependent) endothelialization of the surfaces, with poor ECFC confluency, limited cell-cell contacts, and therefore limited expression of CD31 and VE-cad. To the contrary, a confluent endothelium was observed on the hTEMs (Figures 2C,F,I), with ECFCs displaying the typical cobblestone morphology and expressing the characteristic endothelial cell-cell contact proteins VE-Cad (Figure 2C) and CD31 (Figure 2F). Similar results were observed for hTEMs seeded with control HUVECs (Supplementary Figure S2A).

### Characterization of Endothelial-Colony-Forming Cells Markers Expressed on Human Cell-Derived Tissue-Engineered Extracellular Matrices

**FIGURE 3:**
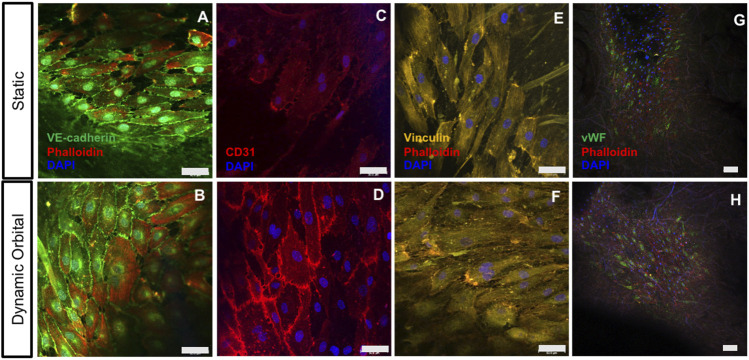
ECFCs cultured on hTEMs express characteristic EC markers after 14 days. **(A–B)**: ECFCs stained for VE-CAD (green), Phalloidin (red), and DAPI (blue) show confluent endothelialization and expression of cell-cell surface markers under both static and dynamic orbital conditions. **(C–D)**: ECFCs stained for CD31 (red) and DAPI (blue) show the expression of ECFCs intercellular junctions under static and dynamic flow conditions. **(E–I)**: Staining for Vinculin (yellow), Phalloidin (red) and DAPI (blue) demonstrating the expression of focal adhesions by ECFCs. **(G–H)**: Staining for vWF (green), Phalloidin (red), and DAPI (blue) demonstrate endothelium functionality (50 µm scale bars).

After 14 days of static or dynamic orbital *in vitro* culture, ECFCs (*n* = 2 donors) seeded on the hTEMs were tested for specific Endothelial cells (ECs) marker expression. ECFCs formed a stable and confluent endothelium retaining the expression of characteristic ECs markers such as VE-cad (Figures 3A,B), CD31 (Figures 3C,D), and vWF (Figures 3G,H). Cell adhesion to the substrate was confirmed by the abundant presence of focal adhesion points as stained by Vinculin (Figures 3E,F) in both static and dynamic culture conditions.

### Scratch Assay and Experimental Crimping Tests

**FIGURE 4:**
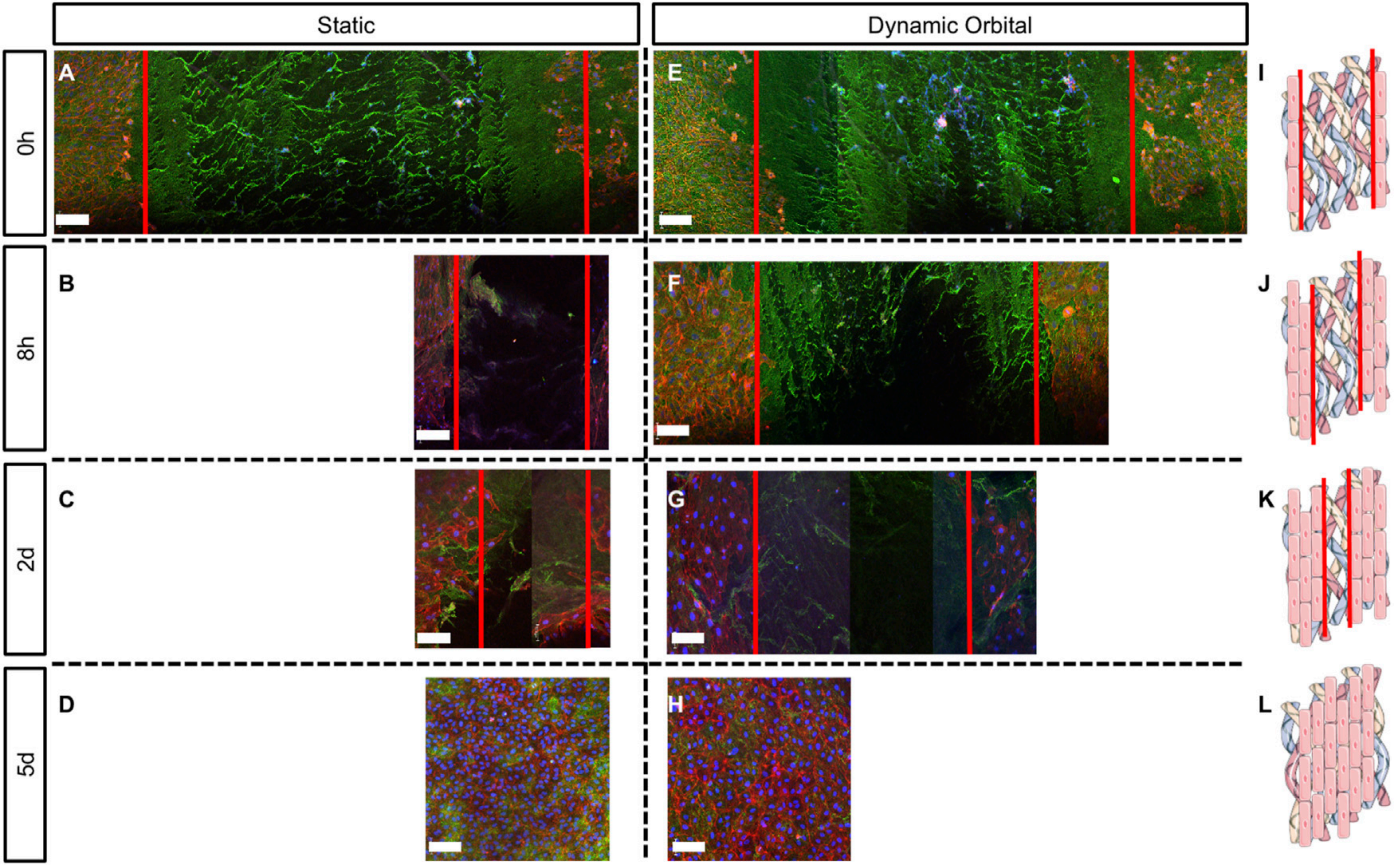
Scratch assay performed on hTEMs seeded with ECFCs and cultured with FBS as medium supplement. Scratch was performed in the middle of the hTEM (red lines). ECFCs were cultured for up to 5 days under static **(A–D)** or dynamic orbital conditions **(E–H)**, and finally stained for Collagen III (green), phalloidin (red) and DAPI (blue) at four different time points (0 h, 8 h, 2 days, and 5 days). **(A,E)**: Control scratch performed at time 0, under static and dynamic orbital conditions, respectively. **(B,F)**: ECFCs migration at 8 h timepoint under static and dynamic orbital conditions, respectively. **(C,G)**: ECFCs migration at 2 days timepoint under static and dynamic orbital conditions, respectively. **(D,H)**: ECFCs reached confluency after 5 days from scratch. **(I–L)**: Schematic representation of the scratch assay test on hTEMs (Images adapted from Servier Medical Art under a creative common attribution 3.0 unported license) (500 µm scale bars).

**FIGURE 5:**
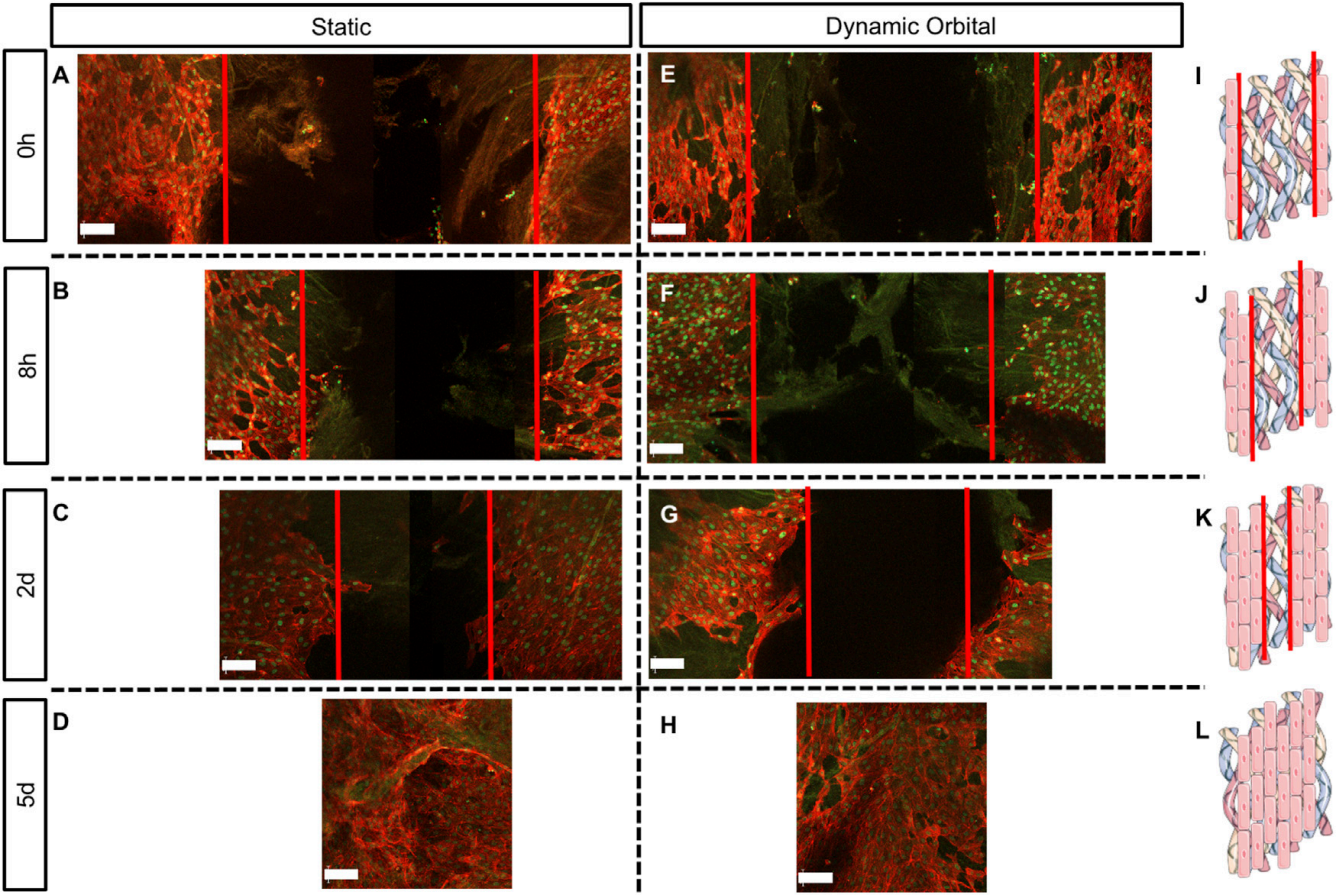
Scratch assay performed on hTEMs seeded with ECFCs and cultured with hPL as medium supplement. Scratch was performed in the middle of the hTEM (red lines). ECFCs were cultured for up to 5 days under static **(A–D)** or dynamic orbital conditions **(E–H)** and finally stained for Collagen III (green), phalloidin (red) and DAPI (blue). **(A,E)**: Control scratch performed at time 0, under static and dynamic orbital conditions, respectively. **(B,F)**: ECFCs migration at 8 h timepoint under static and dynamic orbital conditions, respectively. **(C,G)**: ECFCs migration at 2 days timepoint under static and dynamic orbital conditions, respectively. **(D,H)**: ECFCs reached confluency after 5 days from scratch. **(I–L)**: Schematic representation of the scratch assay test on hTEMs (Images adapted from Servier Medical Art under a creative common attribution 3.0 unported license) (500 µm scale bars).

Scratch assay was used to assess the ability of ECFCs to migrate and restore a confluent endothelium (Figures 4, 5) once seeded onto hTEMs using HUVECs as a control (Supplementary Figure S2B). After performing a damage of about 2 mm in width in the middle of the patches (Figures 4A,E, 5A,E, and Supplementary Figure S2B, red lines), results showed that ECFCs, as well as HUVECs, were able to regenerate the endothelium by restoring its integrity within 5 days of culture with FBS under both static and dynamic orbital conditions (Figure 4). By using hPL as medium supplement, comparable results were observed, demonstrating good migration potential within 5 days after scratch for both static and dynamic orbital conditions (Figure 5). Additionally, ECFCs cultured on hTEMs with hPL as medium supplement were able to spontaneously form microvessel-like structures (Supplementary Figure S3, dotted circles). Importantly, and independently from the culture conditions used, ECFCs were able to restore a homogeneous collagenous matrix composed of Collagen III (Figures 4D–H, 5D–H) and Collagen IV (Supplementary Figure S4).

### Gene Expression Profile of Endothelial-Colony-Forming Cells on Human Cell-Derived Tissue-Engineered Extracellular Matrices

**FIGURE 6:**
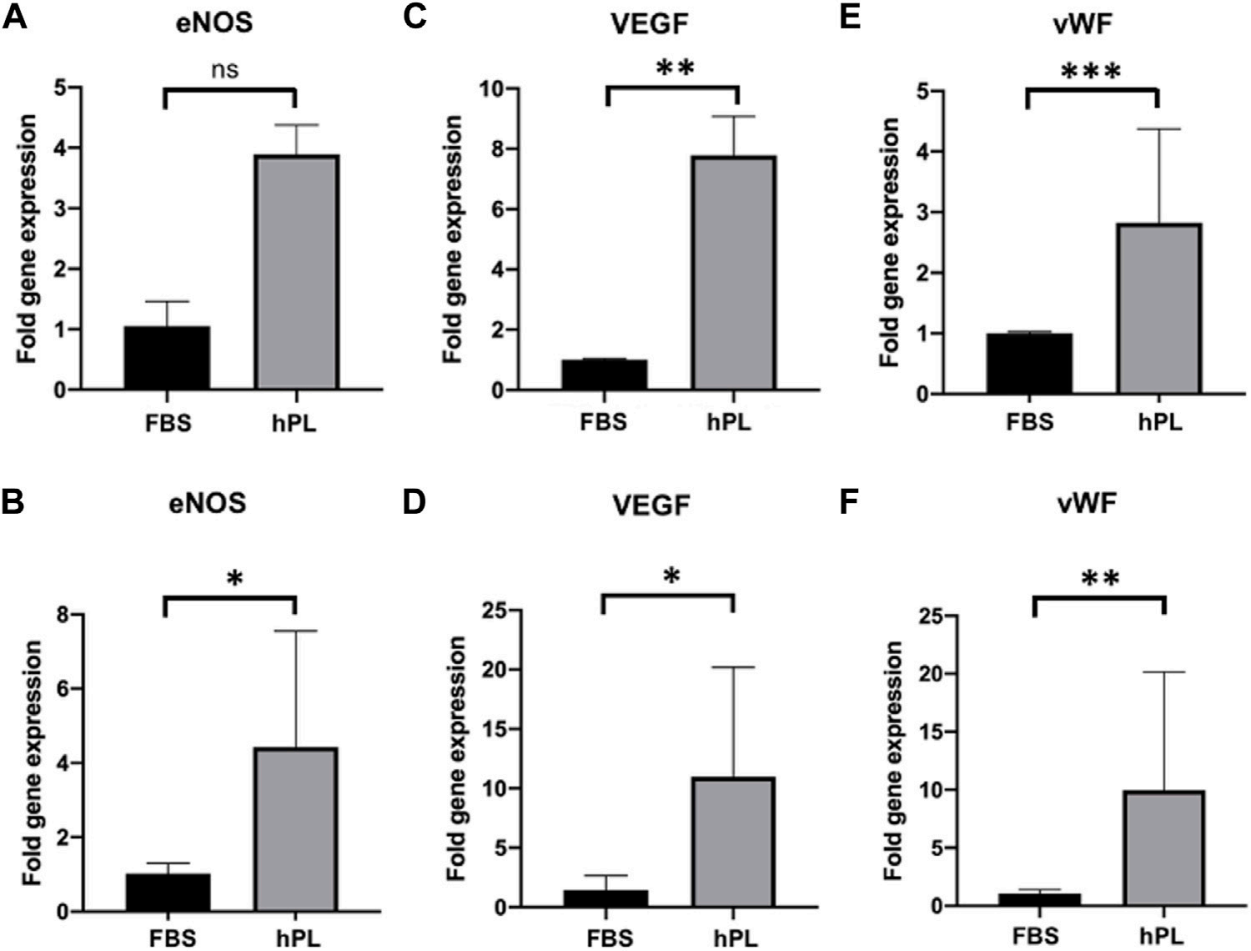
Gene expression profile of hTEMs seeded with ECFCs under static and dynamic orbital conditions using FBS or hPL as medium supplement after 5 days of culture. The gene expression is displayed in dCt and normalized on the average expression level of the housekeeping gene glyceraldehyde-3-phosphate dehydrogenase (GAPDH). **(A,B)**: eNOS expression under static **(A)** and dynamic orbital **(B)** conditions. **(C,D)**: VEGF expression under static **(C)** and dynamic orbital **(D)** conditions. **(E,F)**: vWF expression under static **(E)** and dynamic orbital **(F)** conditions. The cutoff for statistical significance was considered to be *p* < 0.05 (*p < 0.05; **p < 0.01; ***p < 0.001). ns = non-significant.

The gene expression profile of hTEMs seeded with ECFCs and HUVECs under static and dynamic orbital conditions was evaluated *via* rtPCR after 5 days of culture (Figure 6 and Supplementary Figure S5). The reported graphs show differences between the FBS and hPL cultured hTEMs, with a significantly higher expression of ECFCs marker genes such as nitric oxide synthase (eNOS, Figures 6A,B), vascular endothelial growth factor (VEGF, Figures 6C,D) and von Willebrand Factor (vWF, Figures 6E,F) in the hPL cultured group compared to the FBS, under both static and dynamic orbital conditions. For HUVECs gene expression profile more variability was observed depending on the culture conditions used (Supplementary Figure S5). In fact, if cultured under static conditions HUVECs showed comparable expression of eNOS and vWF when supplemented with either FBS or hPL. Remarkably and similarly to ECFCs, expression profiles of the genes eNOS, VEGF, and vWF were upregulated when HUVECs were supplemented with hPL under dynamic orbital conditions (Supplementary Figures S5B,D,F).

### Pre-Endothelialization and Crimping of an Human Cell-Derived Tissue-Engineered Extracellular Matrix-Based Tissue Engineered Sinus Valve

**FIGURE 7:**
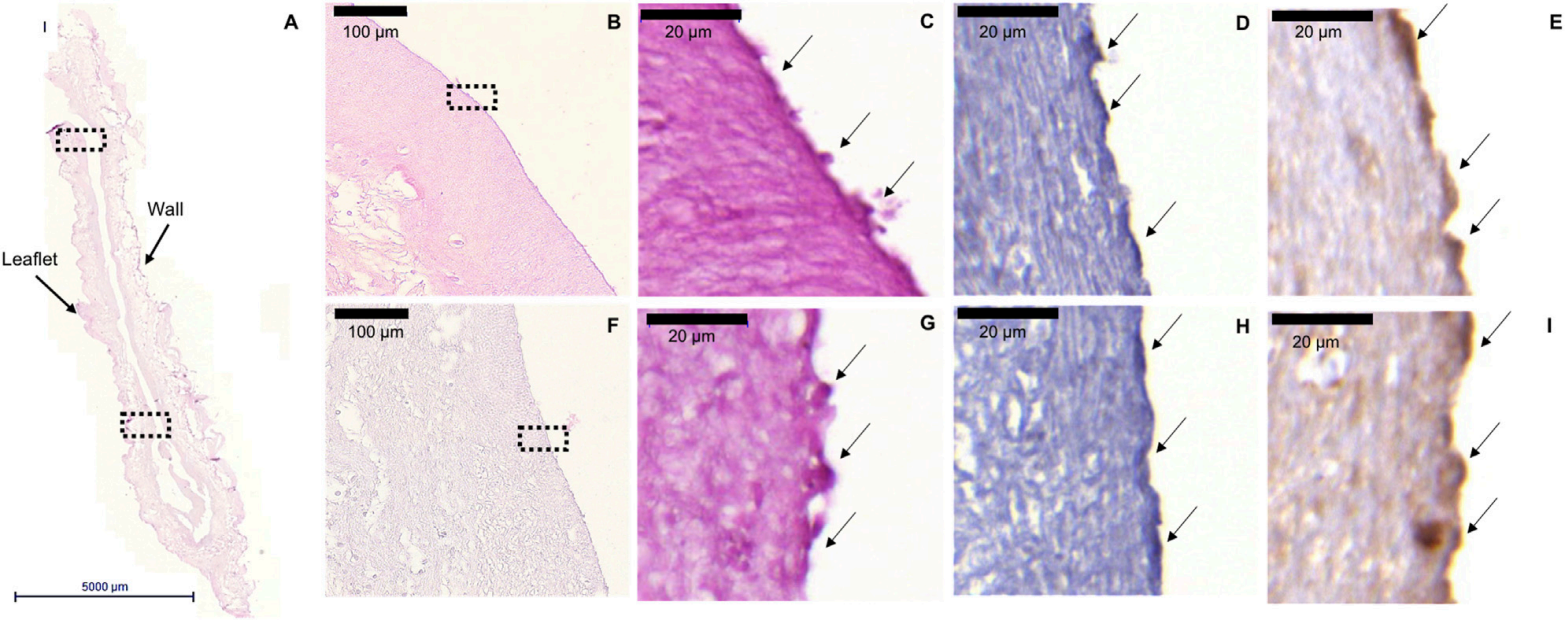
ECFCs retention after TESV crimping procedure. Representative images of the H&E, CD31, and vWF stainings performed on the cross section of the valve. **(A)**: H&E staining shows general morphology of TESV (5000 µm scale bar). **(B,F)**: ×20 magnification pictures of the H&E staining of the upper **(B)** and lower **(F)** leaflet of the valve showing the presence of ECFCs on the surface (100 µm scale bars). **(C,G)**: ×100 magnification pictures of H&E staining of the upper **(C)** and lower **(G)** leaflet marked by the dotted rectangles in panels **(B,F)** respectively (20 µm scale bars). **(D,H)**: ×100 magnification pictures of CD31 staining shows ECFCs presence in the upper **(D)** and lower **(H)** leaflet (20 µm scale bars). **(E,I)**: ×100 magnification pictures of vWF staining expressed by ECFCs in the upper **(E)** and lower **(I)** leaflet (20 µm scale bars).

*In vitro* pre-endothelialization of hTEM-based TESVs (*n* = 4) was confirmed *via* histological analysis (Figures 7A–I). After a crimping procedure of 20 min (Supplementary Figures S6A–F), TESVs morphology was preserved, pre-seeded ECFCs were detected at the valve surface and their presence (H&E and CD31) as well as functionality (vWF) was confirmed by (immuno) histological stainings of the upper (Figures 7B–E) and lower (Figures 7F–I) TESVs leaflet. Control TESVs seeded with HUVECs also showed the presence of ECs (Supplementary Figure S2C), whereas non-seeded TESV displayed an acellular leaflet surface (Supplementary Figures S8A–E).

## Discussion

The hemocompatibility profile of current cardiovascular implants remains a major clinical challenge. To this end, ongoing research studies (e.g., GALILEO and ATLANTIS trials) are evaluating the impact of thrombosis on valve hemodynamics, valve durability, and its progression (Chakravarty et al., 2020). To prevent thromboembolic events related to the implantation of such substitutes, the routine use of anticoagulation or anti-platelet therapies is still required in many patients, while on the other hand such therapies come at a price of a permanently increased risk of bleeding complications (Leiria et al., 2011). In addition, especially in newborns and children, the use of such therapies is limited due to the risk of severe adverse events (e.g., heparin-induced thrombocytopenia) (Dabbous et al., 2014; Newall et al., 2018). Hence, when considering the significant patient population born with congenital cardiovascular malformations requiring treatment with currently-available cardiovascular implants (DiBardino and Jacobs, 2014; Jacobs et al., 2014), this still represents a significant and unresolved clinical problem around the globe. In addition, due to the embryonic toxicity of anticoagulation therapy (e.g., warfarin), also young pregnant women are not eligible for such therapies; and thus cannot be treated with a mechanical heart valve prosthesis (Alshawabkeh et al., 2016; Ayad et al., 2016).

hTEMs may represent a promising option to overcome the limitations of current cardiovascular prostheses. In fact, in numerous animal studies we have shown that (h)TEMs are mechanically mature enough to be safely applied in the cardiovascular system but, on the other hand, are still premature enough to allow for significant recellularization allowing the host cells to invade into their specific niche thereby continuously remodeling the hTEM into native-like tissue. However, despite their strong *in vivo* remodeling potential, the hemocompatibility profile of hTEMs could be further enhanced by using autologous EPC-based *in vitro* pre-endothelialization approaches, which may be particularly beneficial in vulnerable patient cohorts (e.g., patients with diabetes, children, athletes, pregnant women Weber et al., 2012).

A particular focus in our study was to test the feasibility of pre-endothelialize acellular hTEMs in the context of transcatheter heart valve applications as these are particularly exposed to surface damages due to the required crimping maneuver during valve delivery. Our study demonstrates that hTEMs may provide an ideal microstructural basis for the effective and stable *in vitro* formation, retention, and potential repair (in case of damage) of a confluent and functional human EPC-derived endothelium under both, static, and dynamic orbital conditions (e.g., pulmonary arterial shear forces). In our dedicated *in vitro* system, the application of shear forces did not have a negative impact on the protein expression in EPCs (i.e., cell-cell contact proteins, secreted factors, and matrix components). These findings suggest that hTEMs are able to sustain a functional endothelium under dynamic conditions. As suggested by previous studies (Dardik et al., 2005), laminar shear stress is believed to have an atheroprotective effect on ECs, whereas orbital (oscillatory) shear stress often correlates with areas of atherosclerosis. *In vitro* models of “activated” ECs are usually replicated by means of low magnitude oscillatory shear stress platforms, such as an orbital shaker, as the shear stress is not homogeneously applied throughout the culture well. We demonstrated that the hTEM functionally sustains the growth of EPCs when exposed to low shear magnitudes in an orbital shaker. Additionally, recent computational modeling analyses performed on TEMs-based TEHVs demonstrated that increasing pressure and shear stress conditions (i.e., aortic shear forces) corresponded to an improved preservation of valve functionality (Loerakker et al., 2016; Emmert et al., 2018). We could therefore hypothesize, that at higher magnitudes EPCs should positively respond and do not show major signs of activation. Our data further highlight the migration properties of EPCs on hTEMs in an *in vitro* simulated crimping procedure (i.e., scratch assay). In fact, we hypothesize that the particular composition of our hTEM may promote the adhesion properties of EPCs by allowing the expression of cell-matrix contact proteins (e.g., Vinculin), which in turn may enhance cell retention *in situ*. Moreover, vWF is a well-known mediator for inflammation, which in case of pre-endothelialized hTEMs, may be helpful to further stimulate native host cell recruitment *in situ*.

The principal feasibility to pre-endothelialize matrices such as polymers and xenogeneic or allogenic substitutes has been successfully demonstrated in several preclinical and clinical studies (Zilla et al., 1994; Shinoka et al., 1995; Lehner et al., 1997; Cebotari et al., 2006; Hibino et al., 2010; Roh et al., 2010; Quint et al., 2011; Tudorache et al., 2013; Meier et al., 2014; Melchiorri et al., 2015; Theodoridis et al., 2015; Ma et al., 2017; Drews et al., 2020; Schwarz et al., 2021). In comparison to non-endothelialized counterparts, they showed less neointima and thrombus formation. However, on the other hand, human ECs are also known to have limited capabilities to grow and expand across anastomotic regions (Berger et al., 1972; Zilla et al., 2007), thereby limiting the overall *in vivo* endothelialization capacity of tissue-engineered constructs once implanted.

hTEMs and other, conceptually comparable acellular technologies have demonstrated strong over time endothelialization following implantation in recent preclinical studies (Syedain et al., 2017; Biermann et al., 2019)^,^. However, despite this potential, evidence in the human setting remains preliminary (Shirzad et al., 2017) and a systematical investigation in larger clinical studies are needed. Therefore, to date, precautionary anticoagulation or anti-platelet therapy would be still advisable (Lawson et al., 2016; Tudorache et al., 2016; Bobylev et al., 2019; Boethig et al., 2019; Kirkton et al., 2019).

Recently, hPL has been validated and suggested as a clinically relevant alternative to the FBS used for standard cell culture procedures, especially in the setting of wound healing (Bieback, 2013; Martínez et al., 2015; Shirzad et al., 2017). In fact, recent studies reported reduced inflammation, improved bone regeneration, and improved hard and soft tissue wound healing when hPL was added as medium supplement (Chiara Barsotti et al., 2013; Ramos-Torrecillas et al., 2014), further highlighting the potential of hPL for clinical application.

In our study, EPCs pre-endothelialization of hTEMs cultured with hPL demonstrated the capacity to form microvessel-like structures, suggesting the ability of EPCs to rearrange and reorganize to induce angiogenesis (Fortunato et al., 2016). This finding might be attributable to specific factors that are present in hPL such as vascular endothelial growth factor (VEGF, a sub-family of platelet-derived growth factors), which is able to induce the formation of blood vessels (Shanskii et al., 2013; Ramos-Torrecillas et al., 2014). Interestingly, the results presented in this study suggest an anti-thrombotic effect of EPC seeded hTEMs. Here, ECFCs gene expression showed an upregulation of the genes involved in vascular tone control (eNOS), in angiogenesis (VEGF), and hemostasis and EPCs activation (vWF).

Finally, we were able to demonstrate that the concept of EPCs pre-endothelialized hTEMs is also feasible in the setting of a transcatheter heart valve geometry, and in particular, that such pre-endothelialized hTEM-based transcatheter valves can safely undergo crimping maneuvers which are mandatory for transcatheter valve delivery.

This is an important finding, as to date, only surgical tissue-engineered heart valves have been investigated as possible starter matrices for pre-endothelialization studies (Zilla et al., 2008; Stassen et al., 2017). Moreover, due to the increasing clinical relevance of transcatheter valve replacements, their forthcoming suitability for low-risk and young patients (Arora et al., 2017; Kenny and Hijazi, 2017; Fioretta et al., 2018), and our recent advances in the development of transcatheter tissue-engineered heart valves, our results are of high translational relevance, as it is of key importance to understand whether such prostheses could cope with *in vitro* pre-endothelialization procedures**.**

Crimping maneuvers are indeed very frequent “folding techniques” in order to enable transcatheter delivery of cardiovascular prostheses such as stented heart valves, vascular conduits or occluders (Jung and Choi, 2018; Mack et al., 2019). Crimping of current cardiovascular substitutes down to diameters of 14–18 Fr has been demonstrated to cause microstructural damages which could potentially result in an accelerated failure of the prosthesis (Kiefer et al., 2011; Amahzoune et al., 2013; Alavi et al., 2014). Notably, the non-regenerative nature of the fixed xenogeneic bioprostheses may not enable the complete recovery of the initial surface structure, thereby favoring platelets adhesion and long-term calcifications. In our study, we have successfully demonstrated that 1) pre-endothelialized hTEMs can be adjusted to a TESV geometry; and 2) TESVs sustained human EPCs adhesion following the crimping maneuver. Importantly, due to the strong regenerative potential of EPCs together with their migration capacity once seeded onto our hTEMs, we may hypothesize that potential damages that may have occurred during crimping may be repaired *in vivo* following implantation.

## Limitations

This study focused on the *in vitro* evaluation of EPC behavior when seeded onto hTEMs, by means of simplified *in vitro* techniques (e.g., scratch assay). *In vivo* model system showing the direct functionality of pre-endothelialized hTEMs once in contact with animal/human blood was not assessed and was beyond the scope of the study. Future studies should investigate how hTEMs pre-endothelialized with EPCs will impact on the mechanical and functional cardiovascular environment, also taking into consideration blood flow hemodynamics and immune cell response. In this context, in-depth understanding of EPCs anti-thrombotic molecular pathways over time could be analyzed to better predict EPCs behavior *in vivo*. In addition, taking into account the impact of the cell source (i.e., allogenic or autologous) and the existing inter-patient variability (e.g., age, health status, environmental factors) to better understand the dynamic of EPCs behavior *in vivo* and improve their translation to clinical application, an increased patient number could be implemented, also including EPCs isolated from diseased patients.

## Conclusion

This study demonstrates the feasibility to develop an EPCs pre-endothelialized hTEM-based construct as starting matrix for cardiovascular applications. EPCs were successfully retained on hTEMs and showed over time migration properties as well as expression of typical endothelial cells markers such as VE-Cad, CD31, and vWF, under both static and dynamic orbital conditions. The translational potential of such matrices was further proven by the implementation of xenogeneic-free culture protocols and the application of physiological pulmonary shear forces with the characterization of a mature endothelium. Additionally, proof-of-concept pre-endothelialization of clinically-relevant TESVs was successfully demonstrated and the retention of EPCs endured also after crimping procedure, which opens up new avenues towards next-generation pre-endothelialized TESVs, that may provide an enhanced hemocompatibility profile, which may therefore be particularly beneficial in certain indications and for selected patients at high risk for thromboembolic events. Together, our findings provide further important insight into the potential of hTEMs as a next-generation technology for the development of cardiovascular implants.

## Materials and Methods

### Preparation and Culture of Human Cell-Derived Tissue Engineered Matrices

Human cell-based TEMs (hTEMs, *n* = 160) were fabricated using 6 cm^2^ patches of non-woven polyglycolic-acid meshes (PGA; thickness 1.0 mm; specific gravity 70 mg/cm^3^; Cellon, Bereldange) coated with 1% poly-4-hydroxybutyrate (MW 1 × 10^6^: P4HB; TEPHA Inc.) in liquid tetrahydrofuran (Fluka Sigma-Aldrich) (Figure 1A). After overnight drying, the patches were sutured onto stainless steel rings (28 mm in diameter, RVS Paleis) by using continuous sutures (Yavo, PVDF, non-resorbable, 6/0 USP) (Figure 1B). The scaffolds were finally sterilized using a 70% EtOH solution for 30 min and then rinsed twice with phosphate buffer saline (PBS, Sigma-Aldrich) supplemented with 2% amphotericin (Gibco, Invitrogen AG) for 30 min. Finally, scaffolds were incubated overnight at 37°C with advanced Dulbecco’s Modified Eagle Medium (DMEM, Gibco, Invitrogen AG), supplemented with 10% fetal bovine serum (FBS, Gibco, Invitrogen AG), 1% GlutaMax (Gibco, Invitrogen AG), 1% penicillin-streptomycin (Lonza) and L-ascorbic acid 2-phosphate (0.25 mg/ml; Sigma-Aldrich). Human dermal fibroblasts (hDFs) were purchased by CellSystems Biotechnology (Vertrieb GmbH) and expanded in advanced DMEM medium, supplemented with 10% FBS, 1% GlutaMax, and 1% penicillin-streptomycin, and cultured in a cell incubator containing 5% CO_2_ at 37°C. Afterwards, hDFs (1.0 × 10^6^ cells/cm^2^) were seeded onto the scaffolds using fibrin as cell carrier (Mol et al., 2005a) (Figure 1A). Medium was replaced every 2–3 days. After 4 weeks of culturing, patches were decellularized as previously described (Dijkman et al., 2012) (Figures 1A–D). Shortly, patches were washed in PBS and incubated over-night with a detergent solution (PBS supplemented with 0.25% Triton-X-100 (Merck), 0.25% sodium-deoxycholate (Sigma-Aldrich) and 0.02% ethylenediaminetetraacetic acid (Sigma-Aldrich) at 37°C, followed by incubation for 5–8 h with a Benzonase Nuclease (Novagen, Merk) treatment solution (TRIS-HCl buffer, pH = 8) at decreasing concentrations (first treatment: 100 U/ml; second treatment: 80 U/ml; third treatment: 20 U/ml) at 37°C. After washing in PBS, patches were stored at 4°C in DMEM medium.

### Isolation of Endothelial-Colony-Forming Cells

Human peripheral blood mononuclear cells were isolated from 60 ml of blood of two healthy volunteers through gradient centrifugation using Histopaque (Sigma-Aldrich) as separation medium (Fioretta et al., 2012). The cells were isolated under Ethical agreement number 2016-00208 and approved by the cantonal authorities. To obtain ECFCs, peripheral blood cells were seeded onto 0.5% gelatin-coated culture plates and cultured with EGM-2 medium. For the first week after isolation, medium was changed every day. From the second week on, medium was refreshed every 2 days. After 27 days of culture, the first ECFCs colonies were visible, harvested and expanded.

### Fluorescence Activated Cell Sorting Characterization of Endothelial-Colony-Forming Cells

Aliquots of 1 million isolated ECFCs from both donors were characterized by fluorescence activated cell sorting (FACS), and HUVECs were used as control. Briefly, cells were resuspended in cold 1% bovine serum albumin (BSA; pH = 7.0, Axonlab, in solution with PBS) and centrifuged at 300 g for 5 min at 4°C. The supernatant was discarded, and the cells again resuspended into 1% BSA/PBS at 4°C. 1% formaldehyde (FormaFix 4%, EBIS) was then added at the cells and the mixture was incubated for 10 min at 4°C. Cells were subsequently washed with 1% BSA/PBS and finally centrifuged. After resuspension with 1% BSA/PBS, the primary antibody (Pacific blue anti-human CD31 antibody, BioLegend, 2.0 µg per 1 M cells) was added and let incubated at 4°C for 30 min. Finally, ECFCs were again washed, centrifuged, and resuspended with 1% BSA/PBS. FACS was performed using High-end BD SORP flow cytometer (Fortessa, BD Biosciences) and BD FACSDiva software (BD Biosciences). Results were elaborated and analyzed with FlowJo software (FlowJo, LLC).

### Seeding of Endothelial-Colony-Forming Cells and Human-Derived Umbilical Vein Endothelial Cells on Human Cell-Derived Tissue-Engineered Extracellular Matrices

ECFCs (*n* = 2 donors) or HUVECs were expanded in EGM-2 medium supplemented with FBS or human platelet lysate (hPL) and cultured in a cell incubator containing 5% CO_2_ at 37°C. Afterwards, cells were seeded onto hTEMs (*n* = 160, 15′000 cells/cm^2^), glass covered with 0.5% gelatin (*n* = 12, 15′000 cells/cm^2^), and Collagen I gels (Symatese, concentration of 1.5% in PBS) (*n* = 12, 15′000 cells/cm^2^) using pooled HUVECs (Thermofisher) as control. After seeding, cells on hTEMs, glass, and collagen I gels were left for 5 days in the incubator to allow for cell adhesion and endothelial cells (ECs) markers expression.

### Orbital Conditioning, Scratch Assay and Experimental Wound Creation

Seeding of ECFCs (*n* = 40) and HUVECs (*n* = 40) on hTEMs was performed as previously described. From the second day after seeding, hTEMs samples were subdivided into a static (*n* = 20) and a dynamic (orbital shaker, *n* = 20) group for both ECFCs and HUVECs. The orbital shaker was set onto 170 rpm to mimic a pulmonary shear force (19.4 dynes/cm^2^) (Dardik et al., 2005; Kim et al., 2015). These conditions were used to assess hTEMs pre-endothelialization feasibility as well as resistance to shear forces.

Scratch assay (*n* = 4) enabled the analysis of migration and remodeling properties of ECFCs once seeded onto hTEMs. Briefly, after reaching confluency (5 days), an artificial gap (scratch) was performed in the central part of the hTEMs. The scratch was created with a p200 pipet tip as previously described (Liang et al., 2007). ECFCs on hTEMs were cultured under static and dynamic orbital (170 rpm) conditions with EGM-2 medium supplemented with FBS (*n* = 20) or hPL (*n* = 20) at 37°C and finally fixed with 4% formalin after different time-points (0 h, 8 h, 2, 5, and 14 days, duplicates per timepoint). Pooled HUVECs were used as control under the same conditions. For a schematic overview of samples and experimental design see Supplementary Table S1.

### Immunofluorescent Staining

At the terminal time point, cells cultured either on glass, Collagen I, or hTEMs were washed with PBS and fixed in 4% formalin for 15–20 min. Afterwards, cells were permeabilized 10 min with 0.1% Triton-X-100 in PBS, again washed in PBS, and blocked with a blocking buffer composed of 2% BSA in PBS for 1 h. Primary antibody for Vascular Endothelial Cadherin (Ve-Cad), Collagen III, Von Willebrand Factor (vWF), Collagen IV, Phalloidin, and Vinculin (Supplementary Table S2) were solubilized in a 1% BSA/PBS solution and incubated for 1 h at room temperature. After washing with 0.1% Tween-20 in PBS and PBS, samples were incubated with the corresponding secondary antibodies (Supplementary Table S2) for 1 h at room temperature. Finally, cells were again washed with 0.1% Tween-20/PBS and PBS and embedded with the Fluoroshield Mounting Medium with DAPI (Abcam). The stained cells were visualized using a Leica SP8 inverse confocal laser scanning microscope (DMI6000 AFC). Image processing was performed using the LAS AF Lite Microscope software (Leica, version 4.0.11706).

### RNA Isolation and Semi-quantitative Real-Time PCR (qRT-PCR)

ECFCs and HUVECs gene expression cultured in FBS or HPL supplemented medium was analyzed using qRT-PCR. RNA was isolated from hTEMs cultured under static (*n* = 3) or dynamic orbital (*n* = 3) conditions using the RNeasy mini extraction kit (Qiagen). Isolation was done according to the manufacturer’s instructions. RNA concentration was measured on NanoDrop spectrophotometer and diluted with water to obtain the same concentration for all samples. Purified RNA was then transcribed to cDNA (iScript cDNA synthesis kit, BIO-RAD) by mixing it with iScript Reverse transcriptase (1 μl) and 5x iScript Reaction mix (4 μl). Synthesis of cDNA was performed in 20 μl reaction volume in PCR-thermocycler (Biometra, Germany). The reaction protocol consisted of priming for 5 min at 25°C, reverse transcription for 20 min at 46°C, and reverse transcription inactivation for 1 min at 95°C. The relative expression of the genes of interest was quantified by using sequence-specific forward and reverse primers (Supplementary Table S3). Custom-designed primers and probes were synthetized by Microsynth (Balgach, Switzerland) whereas commercially available sets were purchased from Eurofins Genomics (Supplementary Table S3). Glyceraldehyde-3-phosphate dehydrogenase (GAPDH) was used as housekeeping gene. qRT-PCR was performed in 10 μl reaction volume using QuantStudio™ 7 Flex Real-Time PCR System (Applied Biosystems™). The cDNA was diluted 1:10 with DEPC-treated water. A master mix for every primer, composed of Fast SYBR Green (Applied Biosystems™), the diluted primers (0.2 μM) and DEPC-treated water was prepared and mixed in a 384-well plate with 2 μl of cDNA. Samples were tested in triplicate for each gene. The cycling parameters were as follows: initial denaturation at 95°C for 2 min, followed by 40 cycles of 95°C for 10 s, annealing at 60°C for 15 s. Denaturation was then achieved at 95°C for 15 s, extension and read fluorescence was done at 60°C for 1 min. The double ddCT method was used to analyze the data in Excel and GraphPad Prism (Version 8.0).

### Pre-Endothelialization of Tissue-Engineered Sinus Valves

#### Preparation and Culture of Tissue-Engineered Sinus Valves

Trileaflet tissue-engineered sinus valves (TESVs, *n* = 4) were fabricated from non-woven PGA scaffold sewn onto radially self-expandable nitinol stents (CARAG AG), as described elsewhere (Motta et al., 2019). Thereafter, the PGA scaffold was coated with 1% P4HB in liquid tetrahydrofuran, dried overnight and sterilized in ethanol. Meanwhile, hDFs (CellSystems Biotechnology Vertrieb GmbH) were expanded in medium and used as cell source to produce ECM during 4 weeks of *in vitro* culture. 1.0 × 10^6^ cells/cm^2^ were seeded onto the TESV scaffold using fibrin as cell carrier. After seeding the valves were cultured into a Diastolic Pulse Duplicator System, which recapitulates the physiological environment of pulmonary heart valves (Mol et al., 2005b). Finally, TESVs were decellularized, as previously described (Dijkman et al., 2012).

#### Seeding of Endothelial-Colony-Forming Cells and Human-Derived Umbilical Vein Endothelial Cells on Tissue-Engineered Sinus Valves

ECFCs and HUVECs were expanded in EGM-2 medium and cultured in a cell incubator containing 5% CO_2_ at 37°C. Afterwards, cells were seeded onto TESVs (15′000 cells/cm^2^). After seeding, cells on TESVs (*n* = 4) were left for 5 days in the incubator to allow for cell adhesion and growth.

#### Experimental Wound Creation by Crimping Procedure

TESVs were crimped for 20 min in a custom-made crimper device (CARAG AG) mimicking the total crimping time needed during a transcatheter valve implantation procedure (Kiefer et al., 2011; Amahzoune et al., 2013; Alavi et al., 2014). The test was performed in order to determine the damaging impact of such procedure on the endothelium.

#### Histology and Immunohistochemistry

TESVs were fixed in 4% formalin, embedded in paraffin and cut into 5 µm sections. To assess ECFCs and HUVECs presence and tissue composition, sections were stained for Hematoxylin-Eosin, CD31 (Biolegend), and vWF.

#### Statistical Analysis

Data in the text are represented as mean ± SD, unless stated otherwise. ECFCs (*n* = 3) and HUVECs (*n* = 3) gene expression were evaluated with non-parametric unpaired t-test. Prism software version 8 (GraphPad Software, Inc., San Diego, California) was used for the analyses.

## Data Availability

The original contributions presented in the study are included in the article/Supplementary Material, further inquiries can be directed to the corresponding author.

## Abbreviations

BSA, bovine serum albumin; EC(s), endothelial cell(s); ECFC(s), endothelial colony forming cell(s); ECM, extracellular matrix; eNOS, nitric oxide synthase; EPC(s), endothelial progenitor cell(s); FBS, fetal bovine serum; hDF(s), human dermal fibroblast(s); hPL, Human Platelet Lysate; hTEM(s), human-derived tissue engineered matrix(-ces); HUVEC(s), human-derived umbilical vein cell(s); P4HB, poly-4-hydroxybutyrate; PBS, phosphate buffer saline; PGA, polyglycolic acid; TEHV(s), tissue engineered heart valve(-es); TEM(s), tissue engineered matrix(-ces); TESV(s), tissue engineered sinus valve(s); VE-Cad, vascular endothelial cadherin; vWF, von willebrand factor.
