# Supplementary material for: Endothelial Progenitor Cell-Based in vitro Pre-Endothelialization of Human Cell-Derived Biomimetic Regenerative Matrices for Next-Generation Transcatheter Heart Valves Applications
Source: Front Bioeng Biotechnol. 2022 Mar 31;10:867877. doi: 10.3389/fbioe.2022.867877 (PMC9008229; doi:10.3389/fbioe.2022.867877)
Supplement: Supplementary file 1 [file DataSheet1.docx]

***Supplementary Material***

**Endothelial progenitor cell-based in vitro pre-endothelialization of human cell-derived biomimetic regenerative matrices for next-generation transcatheter heart valves applications**

Sarah E. Motta^1,2,6^, Polina Zaytseva^1,6^, Emanuela S. Fioretta^1^, Valentina Lintas^1^, Christian Breymann^3^, Simon P. Hoerstrup^1,2^, Maximilian Y. Emmert^1,2,4,5^

*^1^Institute for Regenerative Medicine (IREM), University of Zurich, Zurich, Switzerland*

*^2^Wyss Translational Center Zurich, University and ETH Zurich, Zurich, Switzerland*

*^3^Obstetric Research-Feto Maternal Haematology Unit, University Hospital Zurich, Zurich, Switzerland*

*^4^Department of Cardiovascular Surgery, Charité Universitätsmedizin Berlin, Berlin, Germany*

*^5^Department of Cardiothoracic and Vascular Surgery, German Heart Center Berlin, Berlin, Germany*

*^6^These authors contributed equally and share first authorship*

**Corresponding author:**

Prof. Maximilian Y. Emmert, MD, PhD

Institute for Regenerative Medicine (IREM)

University of Zurich

Wagistrasse 12

8952 Schlieren, Switzerland

Email: *Maximilian.Emmert@irem.uzh.ch*

Tel.: +41 44 634 56 10

**Supplementary Tables**

**Supplementary Table 1: Overview of the study layout.** Sample numbers are referred to 4 repetitions of the scratch assay (n=4) in duplicates for every condition tested.

| n=4 | Conditioning | | | | Timepoints | Donors |
| --- | --- | --- | --- | --- | --- | --- |
|  | Static | | Dynamic orbital | |  | |
|  | FBS | hPL | FBS | hPL | 0 and 8 hours and  2, 5, and 14 days |  |
| ECFCs | 10 | 10 | 10 | 10 |  | 2 |
| Pooled HUVECs | 10 | 10 | 10 | 10 |  | 1 |

**Supplementary Table 2: List of primary and secondary antibodies used for immunofluorescence analyses.**

| **Staining** | **Primary AB** | **Secondary AB** |
| --- | --- | --- |
| **Ve-Cad (CD144)** | Goat anti-human  (Santa-Cruz) | Donkey anti-goat Alexa 488  (Life technologies) |
| **Collagen III** | Rabbit anti-human  (Abcam) | Goat anti-rabbit Alexa 488  (Life technologies) |
| **vWF** | Rabbit anti-human  (Abcam) | Goat anti-rabbit Alexa 488  (Life technologies) |
| **Collagen IV** | Mouse anti-human  (Abcam) | Goat anti-mouse Alexa 568  (Life technologies) |
| **Phalloidin-TRITC** | - | Alexa 568  (Sigma-Aldrich) |
| **Vinculin** | Mouse anti-human  (Thermo-scientific) | Goat anti-mouse Alexa 568  (Life technologies) |

**Supplementary Table 3: Overview of the primer sequences used for gene expression analysis.**

| **Gene** | **Forward (5’ - 3’)** | **Reverse (5’ - 3’)** | **Producer** |
| --- | --- | --- | --- |
| VEGF | CACCATGCCAAGTGGTCCC | GGAAGATGTCCACCAGGGTC | Eurofins Genomics |
| eNOS | GACCCACTGGTGTCCTCT | CCCGAACACACAGAACCT | Mycrosynth |
| vWF | ACTTCCTTACCCCCTCTGGG | TCCTCGGAGAACCTGGTCAT | Eurofins Genomics |
| GAPDH | GTCAGTGGTGGACCTGAC | ACCTGGTGCTCAGTGTAG | Mycrosynth |

**Supplementary Figures**

**
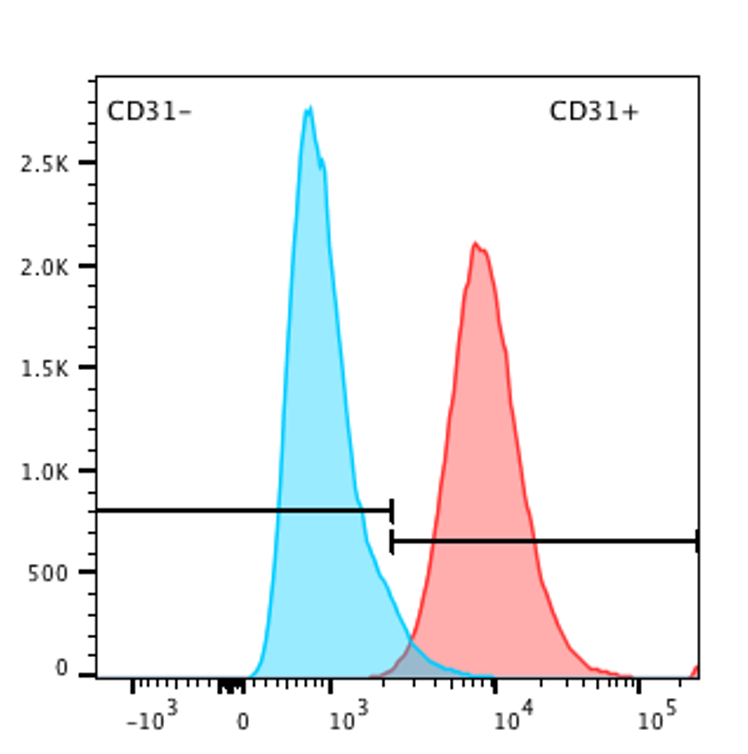
**

**Fig. S1: Representative image of ECFCs expression of the cell marker CD31.**


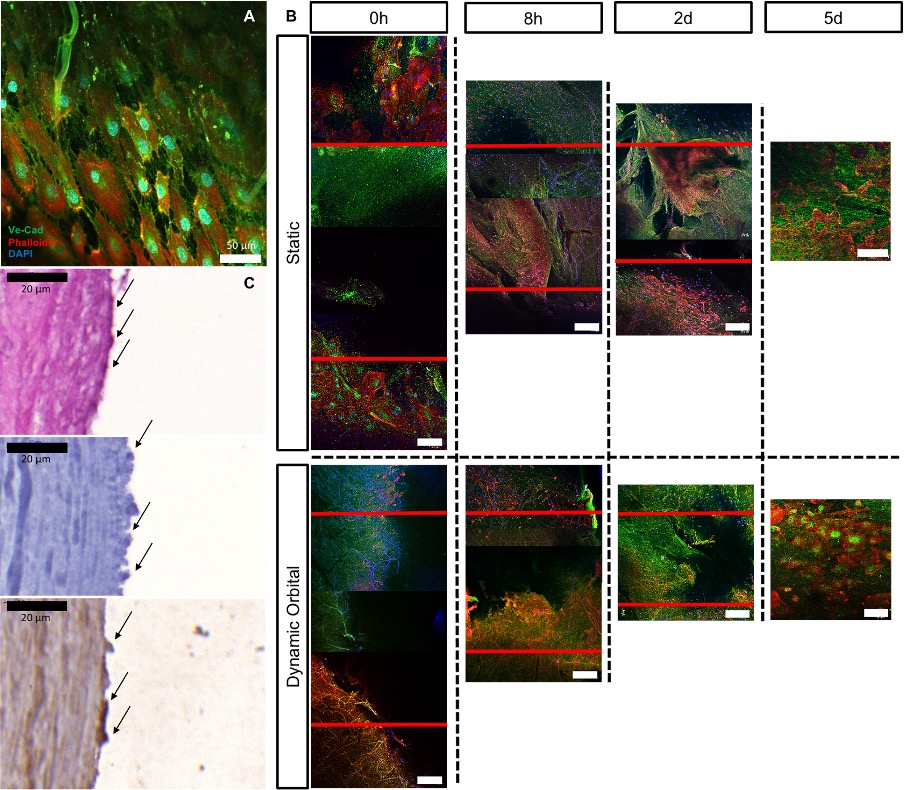


**Fig. S2: Endothelialization potential of HUVECs (control group) on hTEMs**. A: Similar to ECFCs, also HUVECs show pre-endothelialization potential of hTEMs and expression of ECs marker such as VE-Cad (green) (50 µm scale bar). B: Scratch assay performed on HUVECs pre-endothelialized hTEMs shows the migration capacities of HUVECs (500 µm scale bars). C: Application of HUVECs pre-endothelialization on TESVs (20 µm scale bars, 100X magnification).


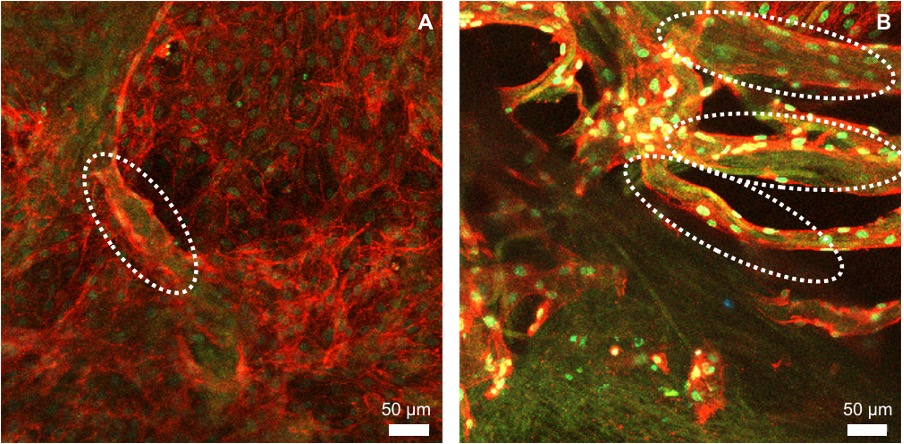


**Fig. S3: ECFCs demonstrate microvessel-like formation after 5 days of culture with hPL as medium supplement.** Representative pictures of ECFCs microvessel-like structure formation (dotted circles) under static (A) and dynamic orbital (B) culture conditions.


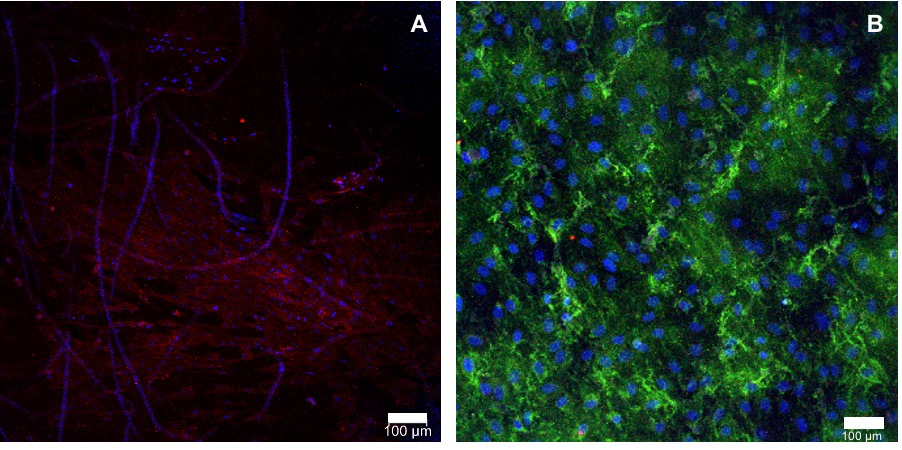


**Fig. S4**: **Expression of basal lamina protein Collagen IV in patches cultured with ECFCs.** A: Control patch showing expression of ECFCs (DAPI, blue and Phalloidin, red) and no expression of collagen IV (green) at day 0. B: Collagen IV (green) is expressed in patches cultured with ECFCs after 14 days.


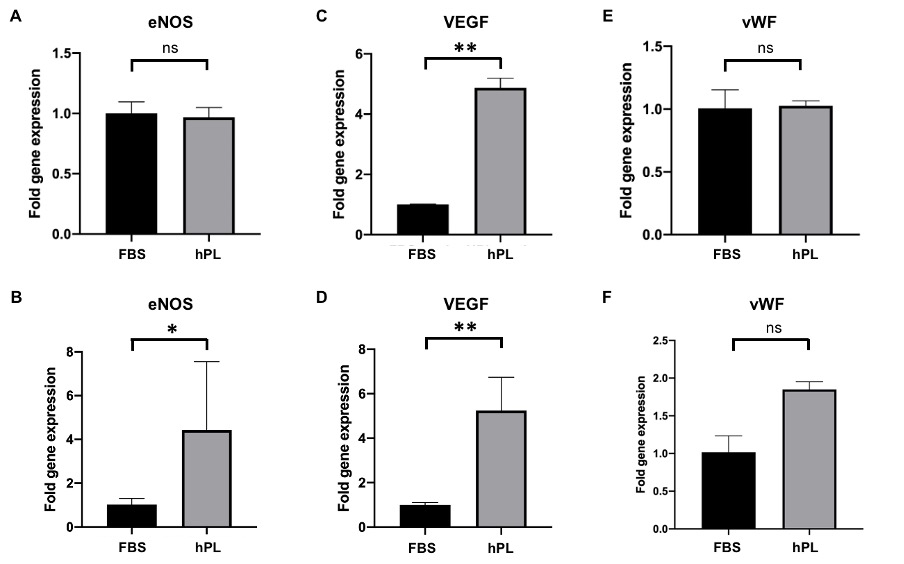


**Fig. S5: Gene expression profile of hTEMs pre-seeded with HUVECs under static and dynamic orbital conditions using FBS or hPL as medium supplement after 5 days of culture**. The gene expression is displayed in dCt and normalized on the average expression level of the housekeeping gene glyceraldehyde-3-phosphate dehydrogenase (GAPDH). A-B: eNOS expression under static (A) and dynamic orbital (B) conditions. C-D: VEGF expression under static (C) and dynamic orbital (D) conditions. E-F: vWF expression under static (E) and dynamic orbital (F) conditions. The cutoff for statistical significance was considered to be p < 0.05 (*p < 0.05; ** p < 0.01; *** p < 0.001). ns = non-significant.


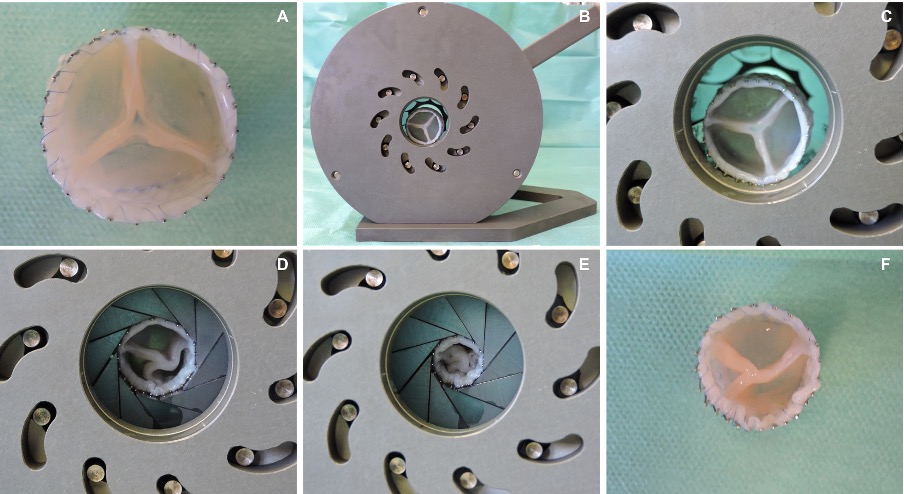


**Fig. S6: Crimping of the TESV.** The TESV undergo a diameter reduction of up to a factor of 2.5 before implantation into the host. A: Appearance of a TESV before crimping. B-C: TESV positioned in the crimper. D-E: Start and end of the crimping procedure. F: Final TESV appearance after crimping.


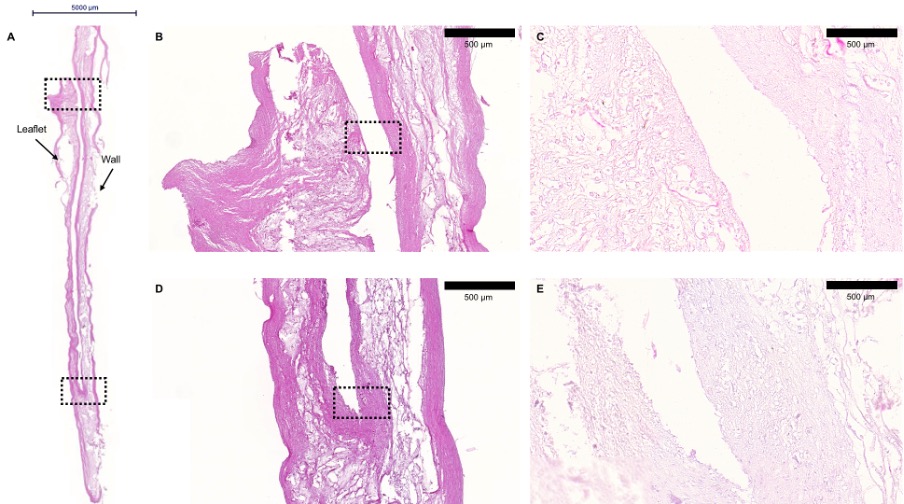


**Fig. S7: Non-seeded TESV after crimping procedure.** Representative images of the H&E stainings performed on the cross section of the valve. A: H&E stainings show general morphology of the TESV (5000 µm scale bars). B and D: 5x magnification pictures of the H&E staining of the upper (B) and lower (D) leaflet of the valve marked by the dotted rectangles in panel A showing an acellular surface (500 µm scale bars). C and E: 20x magnification pictures of H&E staining of the upper (C) and lower (E) leaflet marked by the dotted rectangles in panels B and D respectively (500 µm scale bars).
